# The effect of tetrastarch on the endothelial glycocalyx layer in early hemorrhagic shock using fluorescence intravital microscopy: a mouse model

**DOI:** 10.1007/s00540-022-03138-4

**Published:** 2022-11-24

**Authors:** Tadao Ando, Kohji Uzawa, Takahiro Yoshikawa, Shingo Mitsuda, Yoshihiro Akimoto, Tomoko Yorozu, Akira Ushiyama

**Affiliations:** 1 Department of Anaesthesiology, Kyorin University School of Medicine, 6-20-2 Shinkawa, Mitaka-Shi, Tokyo, 181-8611 Japan; 2 Department of Anatomy, Kyorin University School of Medicine, 6-20-2 Shinkawa, Mitaka-Shi, Tokyo, 181-8611 Japan; 3 Department of Environmental Health, National Institute of Public Health, 2-3-6 Minami, Wakou, Saitama, 351-0197 Japan

**Keywords:** Hydroxyethyl starch, Tetrastarch, Glycocalyx, Massive hemorrhage, Fluid therapy, Colloid resuscitation

## Abstract

**Purpose:**

To investigate vascular endothelial dysfunction based on glycocalyx impairment in massive hemorrhage and to evaluate fluid therapy.

**Methods:**

In this randomized controlled animal study, we withdrew 1.5 mL blood and administered 1.5 mL resuscitation fluid. Mice were divided into six groups according to the infusion type and administration timing: NS-NS (normal saline), NS-HES ([hydroxyethyl starch]130), HES-NS, NS-ALB (albumin), ALB-NS, and C (control) groups.

**Results:**

The glycocalyx index (GCXI) of a 40-μm artery was significantly larger in group C than in other groups (*P* < 0.01). Similarly, the GCXI for a 60-μm artery was significantly higher in group C than in NS-NS (*P* ≤ 0.05), NS-HES (*P* ≤ 0.01), and NS-ALB groups (*P* ≤ 0.05). The plasma syndecan-1 concentration, at 7.70 ± 5.71 ng/mL, was significantly lower in group C than in group NS-NS (*P* ≤ 0.01). The tetramethylrhodamine-labeled dextran (TMR-DEX40) fluorescence intensity in ALB-NS and HES-NS groups and the fluorescein isothiocyanate-labeled hydroxyethyl starch (FITC-HES130) fluorescence intensity in NS-HES and HES-NS groups were not significantly different from those of group C at any time point. FITC-HES130 was localized on the inner vessel wall in groups without HES130 infusion but uniformly distributed in HES130-treated groups in intravital microscopy. FITC-FITC-HES130 was localized remarkably in the inner vessel walls in group HES-NS in electron microscopy.

**Conclusions:**

In an acute massive hemorrhage mouse model, initial fluid resuscitation therapy with saline administration impaired glycocalyx and increased vascular permeability. Prior colloid-fluid administration prevented the progression of glycocalyx damage and improve prognosis. Prior HES130 administration may protect endothelial cell function.

**Supplementary Information:**

The online version contains supplementary material available at 10.1007/s00540-022-03138-4.

## Introduction

Hemorrhage is responsible for > 30% of trauma deaths and cases of intraoperative cardiac arrest [1, 2]. Similarly, acute massive hemorrhage occurs in 30% of scheduled surgeries, and the anesthesiologist's treatment for massive hemorrhage can be problematic later [3]. Thus, appropriate infusion management for bleeding can be an issue of life or death.

The type of initial infusion administered during massive bleeding may affect a patient’s prognosis. Colloids, such as HES130 (hydroxyethyl starch 130/0.4/9; Voluven) and albumin, are often administered when the blood pressure is unstable after crystalloid administration. Transfusion therapy is the first choice for patients with severe massive hemorrhage; however, transfusion products can rarely be administered immediately in clinical situations. A mixture of crystalloids and colloids is routinely used in cases of massive bleeding; however, there is insufficient evidence regarding which infusion product is most effective at reducing mortality.

In a recent study, binding fluorescent dye to HES70 identified its localization in the endothelial surface layer (ESL) of the glycocalyx (GCX) [4], visualizing the direct protective effect of HES70 on GCX under severe conditions; however, HES130 was used for resuscitation and the direct effect of HES130 administration on GCX was not clearly demonstrated.

Circulating blood is composed of two components: a circulating and a non-circulating volume [5]. Pries et al. considered the non-circulating volume to be the immobile plasma layer, and this combined immobile plasma layer and GCX scaffold has been termed “the endothelial surface layer” [6]. GCX generates a passive permeability barrier by creating a scaffolding on which serum proteins are absorbed, forming a gel-like layer on the vascular wall [7]. GCX has garnered increasing attention due to its involvement in fluid management; it plays an important role in maintaining vascular wall integrity and preventing plasma leakage [8].

Depending on the severity of the shock and the type of transfusion therapy, vascular dysfunction has been reported to progress after the loss of blood volume is recovered [9]. Endothelial dysfunction is a major cause of hemodynamic failure and secondary organ dysfunction. GCX is a target for successful resuscitation aftershock [10, 11]. Therefore, minimizing GCX disruption and preserving microcirculation is vital [12].

Hemorrhagic shock can impair endothelial function and induce hyperpermeability, leading to a poor prognosis [13]. Shock-induced endotheliopathy occurs in the early stages of massive hemorrhage [13, 14]. Catecholamine-induced damage to the endothelium causes endothelial breakdown, resulting in GCX shedding and subsequent tight junction deterioration and causing capillary leakage [13]. Additionally, increased expression of disintegration markers in the GCX is associated with increased mortality in trauma patients [15]. Syndecan-1 and hyaluronic acid, important components of GCX, are released into the bloodstream of severe trauma patients due to increased permeability and low plasma colloid osmotic pressure [16, 17]. In patients with massive hemorrhage, fresh frozen plasma can suppress GCX degradation [18, 19]; however, these transfusion products are not always readily available [9, 20].

HES130 can protect the GCX [4, 8, 21, 22]; prevent vascular hyper-permeability [8]; and reduce syndecan-1, heparinase, and hyaluronic acid levels [21]. In 2013, the U.S. Food and Drug Administration and European Medicines Agency cautioned against administering HES130 to critically ill patients due to its potential side effects (renal dysfunction and coagulation disorders). However, recent randomized controlled trials have demonstrated no adverse effects of HES130 infusion on renal function in perioperative patients [23, 24]. It is unclear whether HES130 is effective as a fluid solution in acute hemorrhage, and its suitability for this remains contentious [25].

We predict that prior HES130 administration would attenuate GCX injury during the early stages of acute hemorrhage. HES130 localization in the inner vessel wall can preserve GCX thickness [4, 22]. Therefore, if there is a direct protective effect of HES solutions during massive hemorrhage, prior HES130 administration should be more effective than its later administration in maintaining endothelial function and the GCX layer.

This study aimed to investigate the efficacy of prior HES130 administration on GCX under severe hemorrhagic conditions using mouse dorsal skinfold chambers (DSCs) by intravital microscopy [26] with fluorescent-labeled lectin to visualize the endothelial GCX [27] in vivo. The primary aim was to evaluate GCX thickness, syndecan-1 concentrations, and syndecan-1 immunohistochemical localization on GCX. The secondary aim was to evaluate vascular permeability using tetramethylrhodamine (TMR)-labeled dextran (molecular weight, 40 kDa; DEX40) and fluorescein isothiocyanate (FITC)-labeled HES130 (molecular weight, 130 kDa; HES130). Additionally, we performed blood gas analyses (BGA) and noted the cumulative seven-day mortality of the mice.

## Methods

### Materials

Albumin was purified from mouse serum (Kohsin Bio Ltd., Saitama, Japan) using the modified Cohn method [4] and then eluted from the column using 25 mM acetate buffer (pH 4.5) and neutralized to pH 7.2. Albumin was concentrated in centrifugal filter units with a nominal molecular weight limit of 50 kDa (Amicon Ultra-15 Centrifugal Filter Units; Merck, Darmstadt, Germany). Albumin purification was confirmed by sodium dodecyl sulfate–polyacrylamide gel electrophoresis (SDS-PAGE). The protein concentration was determined using the Bradford Ultra Total Protein Quantitation Kit (Abcam, Cambridge, UK) and adjusted to 5% (w/v) for administration. The albumin solution was stored at − 30 °C until further use.

HES powder (130/0.4/9; average molecular weight: 130 kDa; Fresenius Kabi, Bad Homburg, Germany) was used. HES labeled with FITC was prepared as previously described [4]. Low molecular weight (< 3 kDa) HES fractions were removed using centrifugal filters (Amicon Ultra-15 Centrifugal Filter units, Merck).

### Ethics

Approval for this study (Ethical Committee for Animal Experiments, protocol number: 30–006) was obtained from the Ethical Committee of the National Institute of Public Health, Saitama, Japan, on 5 June 2018.

### Animal preparation

Male BALB/c mice were purchased from Japan SLC Inc. (Shizuoka, Japan). The mice were fed a standard pellet diet (FR-2; Funabashi Farm Co., Chiba, Japan), and their water was acidified with hydrochloric acid ad libitum. The mice were kept in a Super Mouse 1400TM Micro-Isolator Rack (Lab Products, Inc., Seaford, DE, USA) with an artificial 12 h light cycle. All animal experiments were performed and reported in accordance with the Animal Research Reporting of In Vivo Experiments (ARRIVE) guidelines. A dorsal skinfold chamber (DSC) [26] was used to observe microcirculation in the living environment. When the mice were approximately 15 weeks old and weighed 25 g, DSCs were surgically implanted by sandwiching a double layer of the dorsal skin and then removing one side to observe the microcirculation (Online Resource 1) [26]. During surgery, mice were anesthetized using a mixture of ketamine (90 mg/kg) and xylazine (10 mg/kg). They were acclimatized for one week before the experimental procedure. Detailed data regarding the mice are shown in Online Resource 2.

### Experimental protocol

**Fig. 1:**
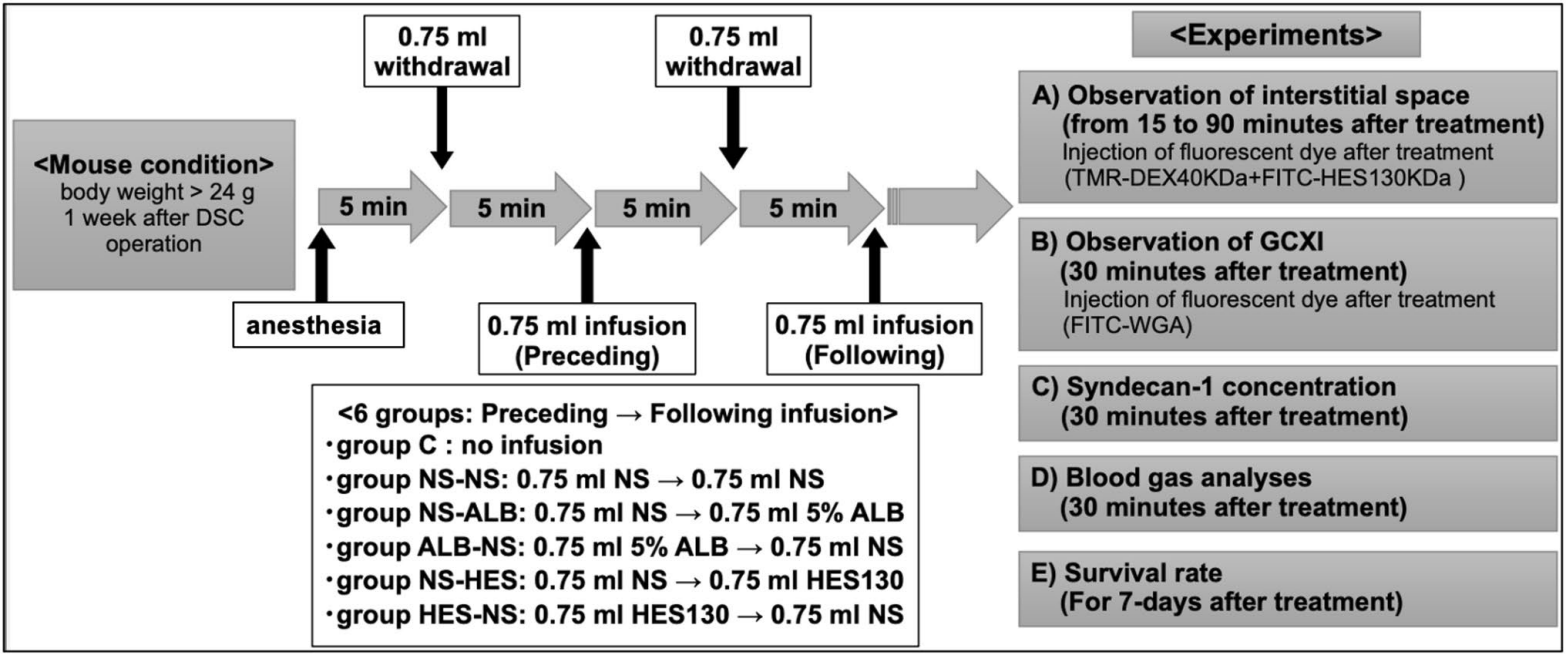
Experimental protocol A dorsal skinfold chamber (DSC) was surgically implanted into approximately 15-week-old male BALB/c mice under anesthesia. At least 1 week after the DSC operation, the mice were anaesthetized again, and the acute hemorrhage experiment was performed as follows: 0.75 mL blood was withdrawn via the jugular vein at a rate of 0.1 mL per 3 s, after 5 min, an equal amount of the allocated fluid in each group was administered. This procedure was then repeated once for a total blood loss of 1.5 mL, with 1.5 mL fluid resuscitation. The mice that underwent blood withdrawal and resuscitation were divided into six groups according to the type of infusion and the timing of administration as shown in the flow chart. After 5 min, the mice were injected with tetramethylrhodamine-labeled dextran (TMR-DEX40) and fluorescein isothiocyanate-labeled hydroxyethyl starch (FITC-HES130), and their fluorescence intensities were measured at regular intervals from 15–90 min (Experiment A). Fluorescein isothiocyanate-labeled wheat germ agglutinin (FITC-WGA) was administered to separate groups of mice treated using the same protocol to calculate the glycocalyx index (GCXI) 30 min after administration (Experiment B). The same treatment was performed on all mice. Thirty minutes after treatment, the blood concentration of syndecan-1 was measured (Experiment C), and a blood gas analysis was performed (Experiment D). The prognosis of the mice was observed for seven days (Experiment E). NS, normal saline; ALB, albumin; HES130, hydroxyethyl starch 130 (Voluven^®^)

An infusion-resuscitation model was constructed after massive bleeding. After anesthesia administration, a catheter was inserted through the internal jugular vein and 1.5 ml of blood was withdrawn twice (70% of the total blood volume). The first dose was administered after a 0.75 ml blood withdrawal, and the second dose was administered after the second withdrawal. Mice were divided into six groups based on the type of first and second doses administered: group NS-NS (normal saline [NS] → NS), NS-HES (NS → HES130), HES-NS (HES130 → NS), NS-ALB (NS → albumin), and ALB-NS (albumin → NS), and group C (no withdrawal and no infusion, only anesthesia; Fig. 1).

### GCX thickness and evaluation of vascular permeability by fluorescent dyes

**Fig. 2:**
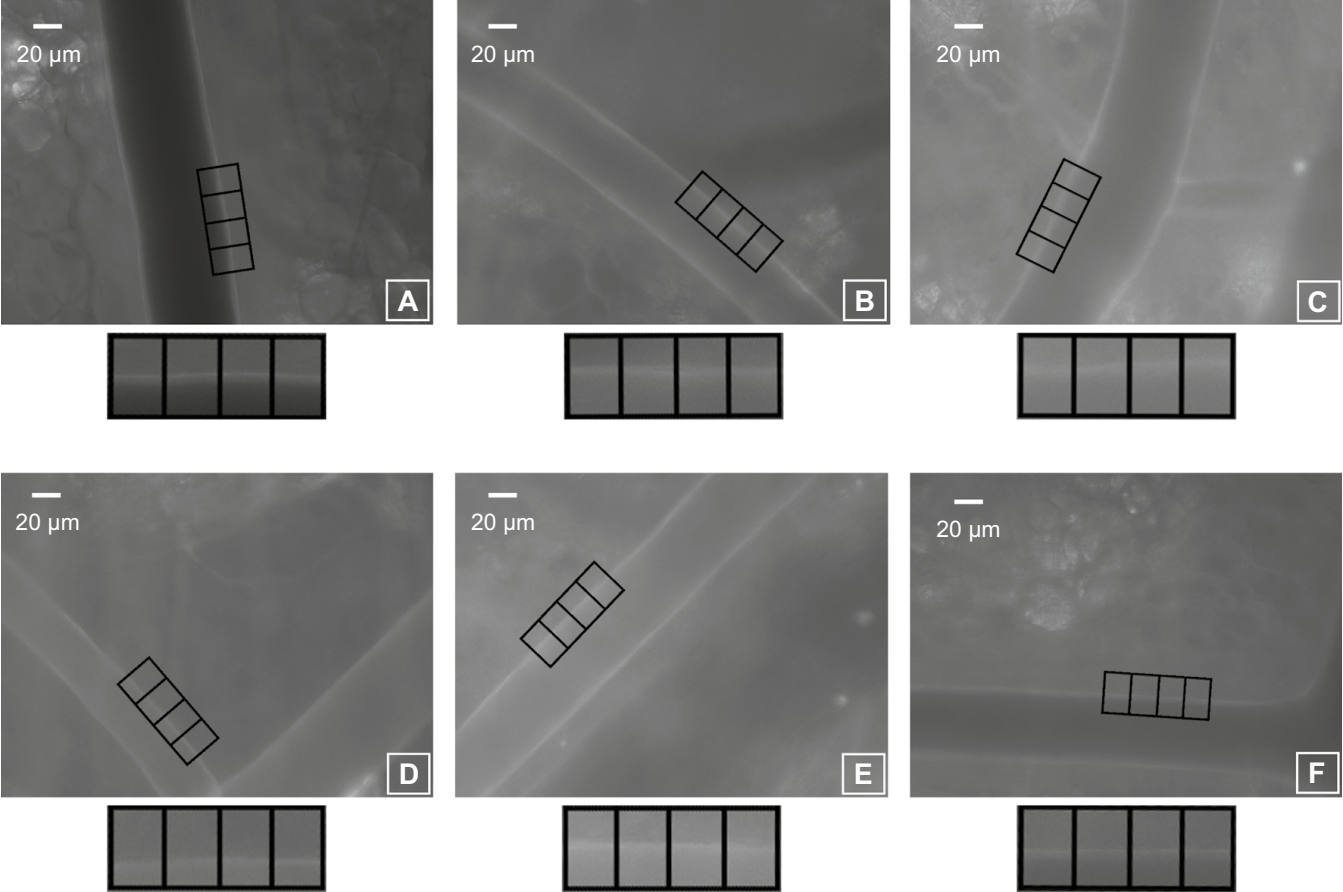
Glycocalyx illumination by FITC-WGA (wheat germ agglutinin) After withdrawal and fluid resuscitation, FITC-WGA was administered through the internal jugular vein and observed 30 min later using an intravital microscope. The inner vessel walls are illuminated as white. Four image blocks were drawn perpendicular to the arterial vessel wall with diameters of approximately 40 μm and 60 μm. **a**: group C, **b**: group NS-NS, **c**: group NS-ALB, **d**: ALB-NS, **e**: group NS-HES130, **f**: group HES130-NS

**Fig. 3:**
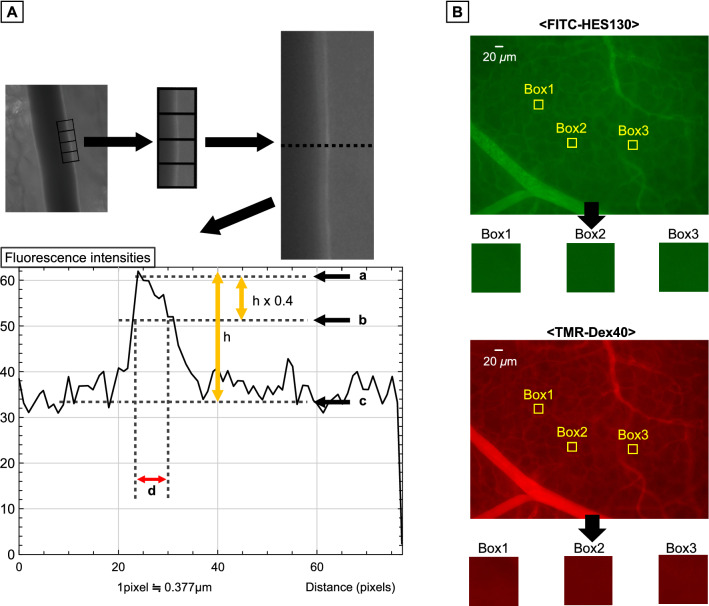
Method for measurement of the glycocalyx index (GCXI) and the fluorescence intensity of interstitial tissue a: Method used to investigate GCXI. To identify curved or overlapping arteries, we drew three perpendicular lines every 50 pixels against one side of the GCX-illuminated vessel wall. The fluorescence intensity on each line was measured, and the pictograms were constructed. The maximum fluorescence intensity was defined as (a), the left and right inflection points were calculated, and the midpoint of these two points was the baseline (c). The distance between two intersections of a straight line (b) and a curve in the upper 40% of (a)–(c) was defined as the GCXI (d). The average of the three GCXIs obtained from a single artery was defined as the GCXI of the individual values. **b**: Quantification of the interstitial fluorescence intensity. After withdrawal and resuscitation treatment, TMR-DEX40 (tetramethylrhodamine-labeled dextran, 40 kDa) and FITC-HES130 (fluorescein isothiocyanate-labeled hydroxyethyl starch 130 kDa) were administered. TMR-DEX40 and FITC-HES130 stained red and green, respectively. We chose areas without surrounding blood vessels and randomly selected three 80 × 80 pixels square areas (boxes 1, 2, and 3). We then measured the fluorescence intensity of TMR-DEX40 and FITC-HES130. The average of the three fluorescence intensity data was defined as the fluorescence intensity at that time, and the changes in fluorescence intensity in the interstitial tissue after 15, 30, 60, and 90 min were observed over time

After treatment, both TMR-DEX40 and FITC-HES130 were injected into the mice via a catheter to evaluate vascular permeability. To estimate the GCX thickness, we used FITC-labeled wheat germ agglutinin (FITC-WGA; Sigma Aldrich, St. Louis, MO, USA). After 30 min, we randomly selected up to three arteries with an inner vessel diameter of 40 ± 10 or 60 ± 10 μm from each chamber, with the walls clearly illuminated by FITC-WGA (Fig. 2). Whenever possible, overlapping and curved arteries were not included in our analysis to prevent potential staining enhancement. We drew three perpendicular lines forming four image blocks over a selected arterial wall, and their fluorescence intensities were measured using ImageJ software (National Institutes of Health, Bethesda, MD, USA) Fig. 3A. The GCX index was measured as previously reported [4, 27, 28]. The average of these values was defined as GCXI. Interstitial fluorescence intensity and GCXI were compared among all groups. Vascular permeability was evaluated using the average fluorescence intensities of TMR-DEX40 and FITC-HES130 in the interstitial tissue. We randomly selected three 80 × 80-pixel square areas (Fig. 3B) without any blood vessels to measure the vascular permeability over time (after 15, 30, 60, and 90 min).

### Measurement of CD138 (syndecan-1) blood concentration

Blood was collected 60 min after treatment, and the plasma concentration of syndecan-1 was measured using the sCD138 ELISA Kit (Diaclone SAS, Besançon, France). The absorbance of the final samples was measured at 450 nm using a microplate reader (Bio-Rad Laboratories, Hercules, CA, USA).

### Immunohistochemical staining for CD138 in cutaneous tissue

CD138 was identified immunohistochemically in paraffin-embedded skin sections using a purified anti-mouse CD138 (syndecan-1) antibody (BioLegend, Inc., San Diego, CA, USA). The dorsal skin of the mice (20 × 20 cm) was surgically excised, fixed in 4% paraformaldehyde phosphate buffer solution, and then embedded in paraffin blocks. The blocks were sliced into 4-μm thick slices on a microtome and mounted on glass slides. Following deparaffinization, antigen retrieval and blocking of endogenous peroxidase activity were performed using an antigen retrieval reagent (R&D Systems Inc., Minneapolis, MN, USA) and BLOXALL (Vector Laboratories, Burlingame, CA, USA), respectively. Immunostaining with anti-mouse CD138 antibody was performed using an HRP anti-rat IgG polymer detection kit (ImmPRESS-HRP, Vector Laboratories) and a DAB EqV peroxidase substrate kit (ImmPACT, Vector Laboratories). All sections were counterstained with hematoxylin staining solution (Sakura Finetek Japan Co., Ltd., Tokyo, Japan) and mounted with coverslips.

### FITC-HES130 localization by immunoelectron microscopy

#### In this study, immunostaining for FITC was performed by the pre-embedding method.

In Group C, FITC-HES130 was administered without blood withdrawal, and the other groups were treated with FITC-HES130 after blood withdrawal. One hour later, the dorsal skin of mice was cut into 15 × 15 mm pieces and immersed in 4% paraformaldehyde for 1 h. After fixation, the skin was washed with phosphate-buffered saline (PBS). The excised skin was transferred to 10% sucrose/PBS and was allowed to equilibrate for 1 h. This replacement was continued in a stepwise fashion up to 20% sucrose/PBS. The skin was embedded with Tissue-Tek OCT Compound (Sakura Finetek Japan Co., Ltd. Tokyo, Japan), and sections (thickness, 10 μm) including small vessels were serially cut in a cryostat (CryoStar NX70, Thermo Fisher Scientific Inc., Waltham, MA, USA). These sections were kept on Superfrost™ Plus Microscope Slides (Thermo Fisher Scientific Inc.)

For FITC immunostaining, the frozen sections with specimens were left at room temperature for about 20 min to thaw and then dried with cold air. The sections were sequentially blocked with BloXall (Vector Laboratories) for 60 min following 5% normal rat serum for 60 min at room temperature. Sections were incubated at room temperature with 1:100 dilution of Anti FITC Polyclonal Antibody (rabbit IgG, Thermo Fisher Scientific Inc.) in PBS. ImmPRESS™ Reagent, Anti-Rabbit Ig, Goat, Peroxidase (Vector Laboratories) was then applied for 30 min. After washing sections with PBS three times, FITC staining was visualized with the ImmPACT™ DAB Substrate Kit (Vector Laboratories).

The sections were washed with PBS and fixed in 1% glutaraldehyde/PBS. After washing with distilled water, the sections were osmicated and then dehydrated through a graded series of ethanol and embedded in Epon 812 (TAAB Laboratories Equipment Ltd, Aldermaston, England). Ultrathin sections were cut, slightly stained with lead citrate, and examined using a transmission electron microscope (JEM 1011; JEOL, Tokyo, Japan).

### Measurement of blood gas and seven-day cumulative survival rates

BGA were performed on all mice at 60 min after treatment using the i-STAT 300F Analyser EC4 + , CG4 + (Abbott, Princeton, NJ, USA). The central catheter was removed after treatment without the administration of the fluorescent dye. The wound on the right internal jugular vein was sutured, and hemostasis was confirmed. The mice were then observed routinely for seven days. The seven-day cumulative mortality rate was recorded.

### Data analysis

Statistical tests were performed using the JMP Statistical Analysis Software (SAS, Cary, NC, USA) and PRISM for Mac OS (version 8.4.3, GraphPad Software, Inc., San Diego, CA, USA). Group C was used as the reference group for each observation. All data were analyzed by one-way analysis of variance (ANOVA) followed by Dunne's post-hoc test except for the seven-day cumulative mortality rate. The seven-day cumulative mortality rate was analyzed using the Kaplan–Meier method followed by the log-rank test; values with *P* ≤ 0.05 were considered statistically significant.

## Results

**Fig. 4:**
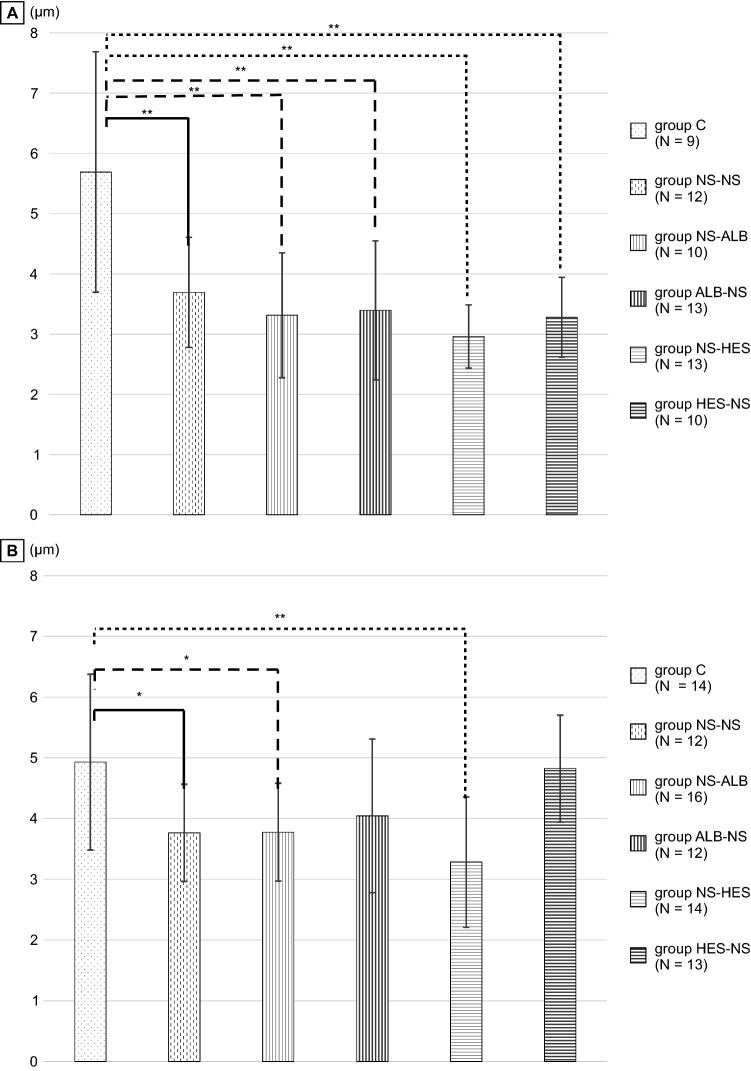
Comparison of the glycocalyx index (GCXI) of each group The fluorescence intensity of the FITC-WGA-positive layer was defined as the GCXI to determine changes in the thickness of the GCX compared with control group C. Figure 4-a shows the GCXI of an artery with a diameter of approximately 40 μm; Fig. 4-b, shows the GCXI of an artery with a diameter of approximately 60 μm. N refers to the number of vessels analyzed in each group. Values are expressed as the mean ± SD. The data were analyzed by one-way analysis of variance (ANOVA) followed by Dunne’s post hoc test. *P < 0.05, compared to group C

The average GCXI in the 40 μm arteries in group C was significantly thicker than that in all other groups (*P* < 0.01; Fig. 4A, Table 1). The average GCXI in the 60 μm arteries in group C was significantly thicker than that in the NS-HES (*P* ≤ 0.01), NS-NS (*P* ≤ 0.05), and NS-ALB (*P* ≤ 0.05) groups. The GCXI of the remaining groups was not significantly thicker than that of group C (Fig. 4B, Table 1).

**Table 1:** Average of FITC-WGA positive layer thickness (glycocalyx index) and syndecan-1 concentration

Group	Group C (*N*)	Group NS-NS (*N*)	Group NS-ALB (*N*)	Group ALB-NS (*N*)	Group NS-HES (*N*)	Group HES-NS (*N*)
40 μm (Vessel diameter: μm)	5.69 ± 1.99 (7)	3.69 ± 0.91^**^ (7)	3.31 ± 1.04 ^**^ (8)	3.40 ± 1.15^**^ (8)	2.96 ± 0.53^**^ (8)	3.28 ± 0.66^**^ (6)
60 μm (Vessel diameter: μm)	4.93 ± 1.45 (10)	3.76 ± 0.80^*^ (9)	3.77 ± 0.80^*^ (8)	4.04 ± 1.27 (7)	3.28 ± 1.07^**^ (10)	4.82 ± 0.88 (8)
Syndecan-1 ng/ml	2.54 ± 0.69 (9)	7.70 ± 5.71^**^ (7)	3.00 ± 0.92 (5)	5.99 ± 3.04 (6)	2.11 ± 0.44 (6)	2.76 ± 1.33 (8)

Groups NS-NS (normal saline (NS) → NS), NS-HES (NS → HES130), HES-NS (HES130 → NS), NS-ALB (NS → albumin), and ALB-NS (albumin → NS), and group C (control, no withdrawal and no infusion). Data are shown as the mean ± SD. *P < 0.05 and ** *P*≦0.01 compared with group C

**Fig. 5:**
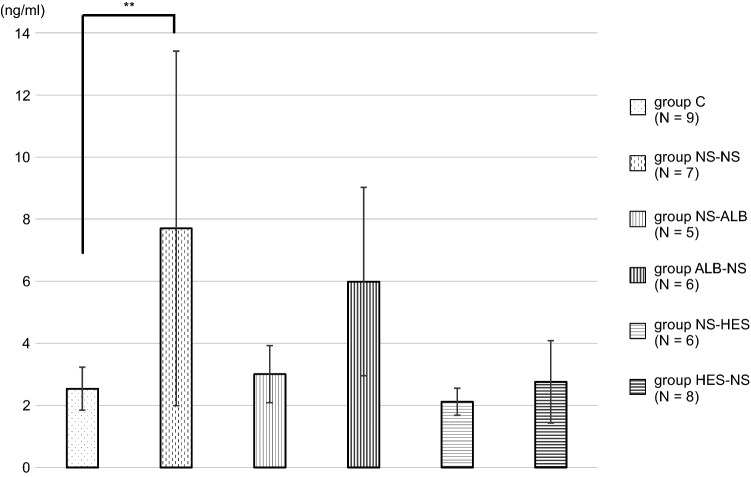
Blood concentration of syndecan-1 Values are expressed as the mean ± SD. The data were analyzed by one-way analysis of variance (ANOVA) followed by Dunne’s post hoc test. *P < 0.05 and ** *P*≦0.01 compared with C

**Fig. 6:**
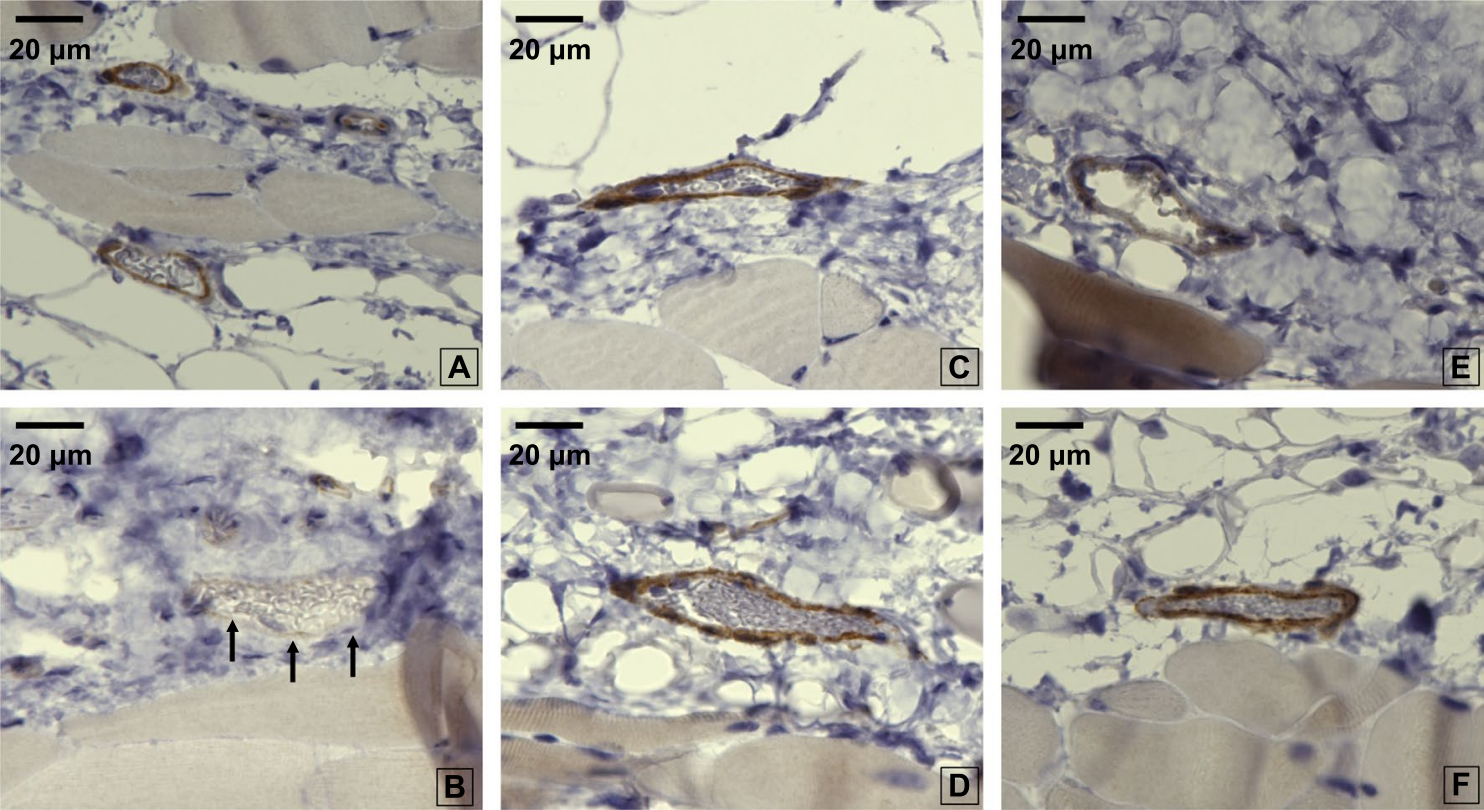
Antibody immunostaining images for syndecan-1 Images of antibody immunostaining for syndecan-1 in all groups. In all images except group NS-NS (**b**), the vessel wall exhibited a strong black stain (black arrows). **a**: Group C (syndecan-1 concentration: 2.580 ng/ml). **b**: NS-NS group (syndecan-1 concentration: 6.301 ng/ml). **c**: NS-HES130 group (syndecan-1 concentration: 1.376 ng/ml). **d**: HES-NS group (syndecan-1 concentration: 0.773 ng/ml). **e**: NS-ALB group (syndecan-1 concentration: 3.042 ng/ml). **f**: ALB-NS group (syndecan-1 concentration: 0.453 ng/ml)

The average concentration of syndecan-1 in group C was significantly lower than that in group NS-NS (7.70 ± 5.71 ng/ml; *P* ≤ 0.01). The average concentrations in the NS-Alb, ALB-NS, NS-HES, and HES-NS groups were not significantly different from those in group C (Fig. 5, Table 1). In the syndecan-1 immunostaining images, the NS-NS group showed little staining of the inner vessel wall when compared to the other groups, with a diameter of approximately 40 µm (Fig. 6).

**Fig. 7:**
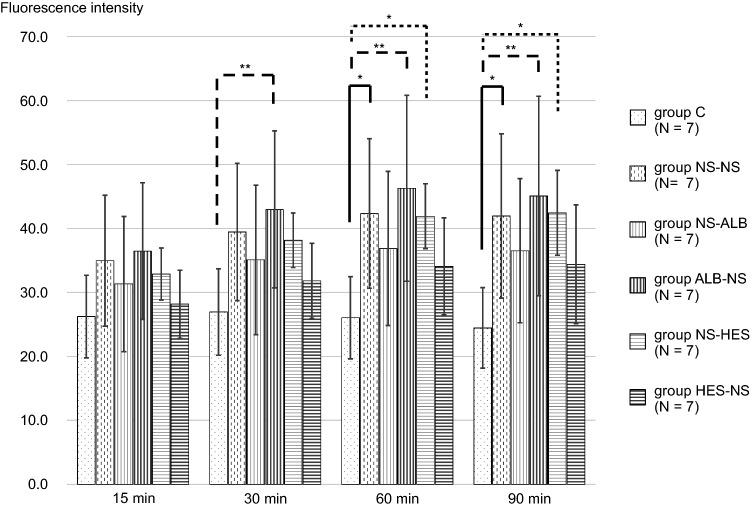
Fluorescence intensity of TMR-DEX40 Changes in fluorescence intensity of TMR-DEX40 (tetramethylrhodamine-labeled dextran: mean molecular weight 40 kDa) in the interstitial tissue were measured over time at 15, 30, 60, and 90 min and compared with those of group C (*n* = 7 for each group). Values are expressed as the mean ± SD. The data were analyzed by one-way analysis of variance (ANOVA) followed by Dunne’s post hoc test. *P < 0.05 and **P≦0.01 compared with C

**Fig. 8:**
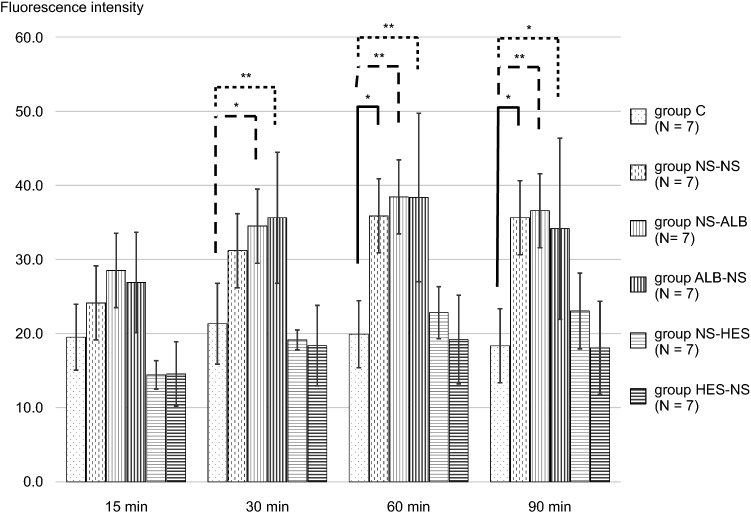
Fluorescence intensity of FITC-HES130 Changes in fluorescence intensity of FITC-HES130 (fluorescein isothiocyanate-labeled hydroxyethyl starch: mean molecular weight 130 kDa in the interstitial tissue were measured over time at 15, 30, 60, and 90 min and compared with group C (*n* = 7 for each group). Values are expressed as the mean ± SD. The data were analyzed by one-way analysis of variance (ANOVA) followed by Dunne’s post hoc test. *P < 0.05 and **P≦0.01 compared with C

**Fig. 9:**
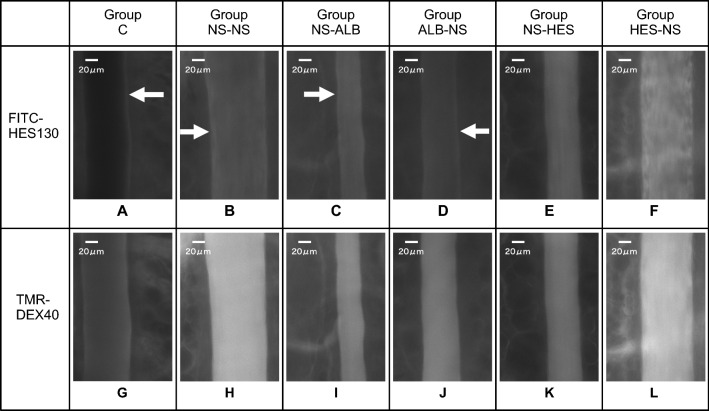
FITC-HES130 localization Representative images at 60 min after treatment in each group. Images a-f are obtained 60 min after FITC-HES130 administration. FITC-HES130 was localized to the intravascular wall (white arrows) in the untreated group C (**a**), NS-NS (**b**), NS-ALB (**c**), and ALB-NS (**d**) groups but was uniformly distributed in the NS-HES (**e**) and HES-NS (**f**) groups. Images G-L were acquired 60 min after TMR-DEX40 administration. All groups (**g-l**) showed uniform intravascular wall staining

**Fig. 10:**
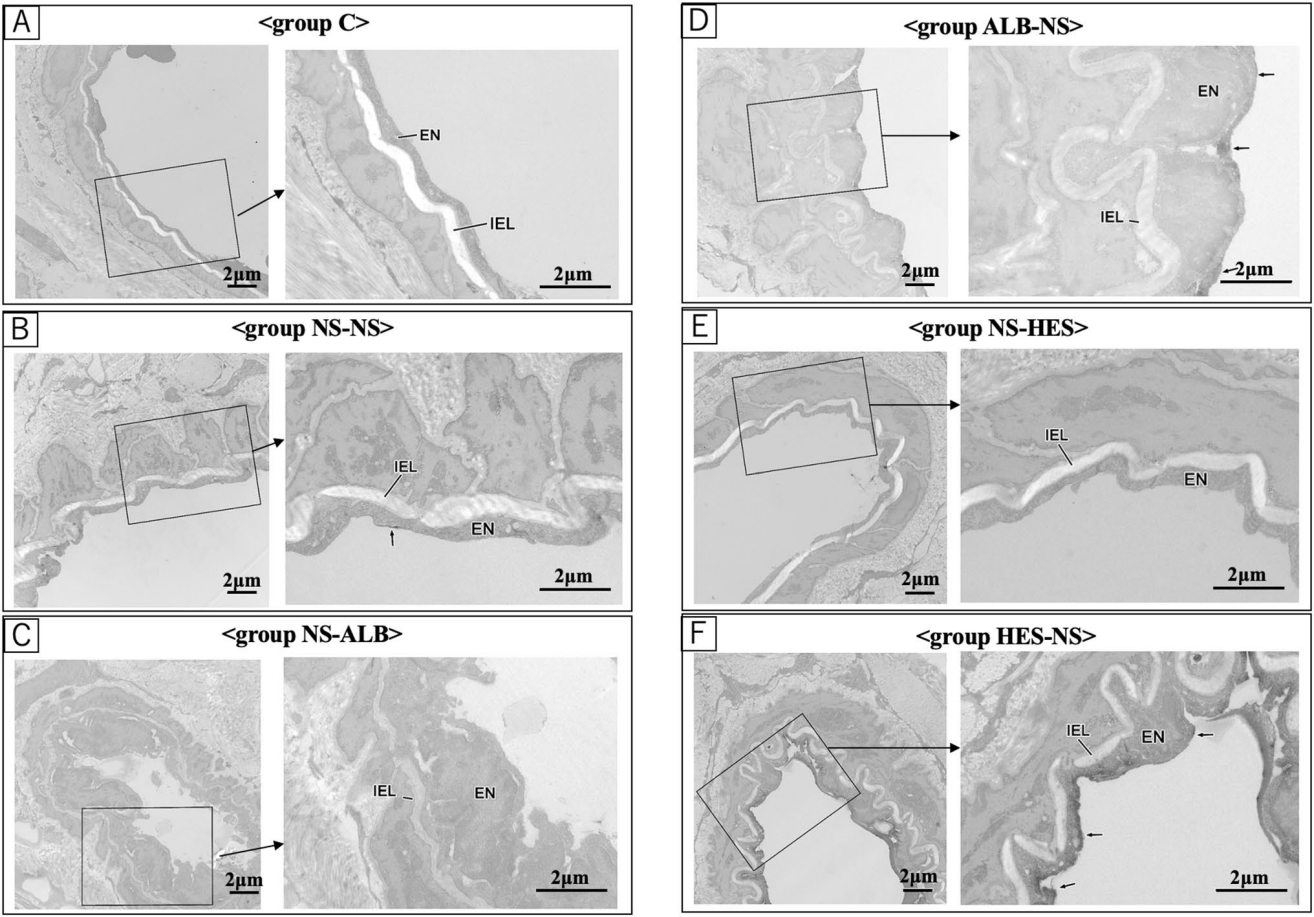
FITC-HES130 localization by immunoelectron microscopy A-F are electron micrographs of the arterial wall of each group. The black arrows ( ←) indicate FITC-HES130-positive areas. Group C, group NS-ALB, and group NS-HES showed almost no FITC-HES130-positive regions (Figure a, c, e). Group NS-NS showed a very weak positive reaction (Figure b), and group ALB-NS showed a positive reaction (Figure d). Remarkably, group HES-NS showed a very intense positive reaction (Figure f). Low magnification, × 3,200; high magnification, × 8,250

The TMR-DEX40 fluorescence intensity in the interstitial space was significantly increased in group ALB-NS when compared with group C (after 30, 60, and 90 min, *P* ≤ 0.01); group NS-NS (after 60 and 90 min, *P* ≤ 0.05); and group NS-HES (after 60 and 90 min, *P* ≤ 0.05). There were no significant differences compared with group C at any time point. The TMR-DEX40 fluorescence intensity in the interstitial space was significantly higher in the NS-NS (after 60 and 90 min); ALB-NS (after 30, 60, and 90 min); and NS-HES (after 60 and 90 min) groups than in group C. There were no significant differences in the NS-ALB and HES-NS groups at any time point when compared to group C (Fig. 7). The FITC-HES130 fluorescence intensity in the interstitial space was significantly higher in the NS-NS (after 60 and 90 min); NS-ALB (after 30, 60, and 90 min); and ALB-NS (after 30, 60, and 90 min) groups than in group C. There were no significant differences in the NS-HES and HES-NS groups at any time point when compared with group C (Fig. 8). In the intravital microscopy image, at 60 min, TMR-DEX40 uniformly stained the intravascular wall in all groups and FITC-HES130 was localized on the inner vessel walls in the groups without HES130 infusion. Simultaneously, FITC-HES130 showed uniform staining in the blood vessels in the groups with HES130 infusion (Fig. 9; Online Resources 3–6). In the immunoelectron microscopy image, FITC-HES130 was strongly localized on the inner vessel walls in group HES-NS (Fig. 10F). It was weakly localized on the inner vessel walls in group ALB-NS and group NS-NS (Fig. 10B, D). There were no FITC-HES130-positive regions in group C; group NS-ALB; or group NS-HES (Fig. 10A, C, E).

In the BGA (Table 2), the pH was significantly lower in all groups than in group C. In contrast, there were no significant differences in PaCO_2_ and PaO_2_ between group C and the other groups. The lactate level showed no significant difference in group NS-HES (1.8 ± 1.71 mmol/L) and group HES-NS (1.2 ± 0.56 mmol/L) but was significantly higher in group NS-NS (4.2 ± 0.99 mmol/L, *P*≦0.01); group NS-ALB (2.9 ± 1.34 mmol/L, *P*≦0.01); and group ALB-NS (3.9 ± 1.37 mmol/L, *P*≦0.01) when compared with group C (0.8 ± 0.51 mmol/L). Hemoglobin levels were lower in all other groups than in group C (*P* ≤ 0.01). The seven-day cumulative mortality was significantly lower than that of group C (100%) in the NS-NS (20%, *P* ≤ 0.01) and NS-HES groups (60%, *P* = 0.0289). There were no significant differences in the remaining groups when compared with group C (Online Resource 7).

**Table 2:** Blood gas analysis parameters

Group Number	Group C (*N*)	Group NS-NS (*N*)	Group NS-ALB (*N*)	Group ALB-NS (*N*)	Group NS-HES (*N*)	Group HES-NS (*N*)
pH	7.172 ± 0.058 (10)	6.980 ± 0.070^**^ (8)	7.068 ± 0.052^**^ (8)	7.037 ± 0.069^**^ (7)	7.088 ± 0.051^*^ (8)	7.077 ± 0.055^**^ (8)
pCO_2_ (mmHg)	83.0 ± 11.73 (10)	85.3 ± 9.85 (8)	82.7 ± 7.19 (8)	86.0 ± 12.10 (7)	85.0 ± 9.96 (8)	87.9 ± 8.41 (8)
pO_2_ (mmHg)	46.1 ± 18.23 (10)	42.3 ± 9.04 (8)	37.6 ± 5.78 (8)	35.7 ± 7.70 (7)	43.0 ± 12.47 (8)	47.8 ± 11.45 (8)
BE (mEq/L)	1.8 ± 1.48 (10)	− 11.5 ± 4.21^**^ (8)	− 6.1 ± 3.44^**^ (8)	− 7.4 ± 4.04^**^ (7)	− 4.4 ± 1.77^**^ (8)	− 4.5 ± 2.00^**^(8)
Lactate (mmol/L)	0.8 ± 0.51 (10)	4.2 ± 0.99^**^ (8)	2.9 ± 1.34^**^ (8)	3.9 ± 1.37^**^ (6)	1.8 ± 1.71 (8)	1.2 ± 0.56(8)
Hb (g/dL)	13.7 ± 1.06 (10)	6.5 ± 1.36^**^ (8)	4.3 ± 1.45^**^ (8)	7.3 ± 0.98^**^ (6)	5.6 ± 1.18^**^ (8)	6.7 ± 0.56^**^ (8)

BE base excess, Hb hemoglobin group NS-NS (normal saline [NS] → NS), NS-HES (NS → HES130), HES-NS (HES130 → NS), NS-ALB (NS → albumin), and ALB-NS (albumin → NS), and group C (control, no withdrawal, and no infusion). Data are shown as the mean ± SD. *P < 0.05 and ** *P*≦0.01 compared with group C

## Discussion

This study aimed to clarify the relationship between HES130 expression and endothelial function in vivo. Additionally, we investigated the effect of different infusion resuscitation regimes with prior administration of HES130 on endothelial dysfunction. Plasma syndecan-1 is a marker for GCX shedding that strongly correlates with reduced GCX thickness and increased microvascular permeability when various fluids are used for resuscitation after hemorrhage [10]. Intravital microscopy has shown that endothelial GCX thickness is reduced after hemorrhage in the skeletal muscle and mesenteric venules [14]. The degradation of endothelial GCX is pertinent to increased GCX fragmentation and fluid resuscitation vascular permeability [9].

The amount of GCX increases in proportion to the vessel diameter and is likely to depend on the vessel size [29]. Moreover, GCX disruption may depend on the vessel size. Previously, Uzawa et al. reported GCX disruption in 20-μm vessels in the massive hemorrhage in a murine model [4]. In our experiment, we focused on changes in GCX present in medium-sized vessels (40–60 μm) due to massive hemorrhage. In this study, prior administration of HES130 into the 40 μm arteries resulted in a significantly lower GCXI among all groups. However, the GCXI of 60 μm arteries after prior administration of HES130 and albumin was not significantly different when compared with group C (Fig. 4A, B). The prior administration of HES130, unlike that of albumin, did not result in significantly higher fluorescence intensity of TMR-DEX40 and FITC-HES130 in the interstitial tissue at 30, 60, or 90 min (Fig. 7, Fig. 8). Prior administration of HES130 suppressed vascular hyperpermeability and was associated with significantly lower lactate levels (Table 2) and no decrease in the seven-day survival period (Online Resource 7), suggesting that prior administration of HES130 during the early stage of massive hemorrhage may inhibit GCX disruption and reduce endothelial dysfunction.

The GCX exists as an ESL with a water-rich gel layer on the superior surface of the inner vessel wall in vivo, and its volume can exceed 1,000 mL in humans [6]. Therefore, when observing the GCX in a biological environment, it is necessary to include this water-rich gel form. Ebong et al. reported that GCX thickness was 3.6–12.5 μm, measured from flash-frozen specimens without dehydration [30]. The thickness of GCX observed in vivo depends on vessel diameter and pathologic environmental factors, such as sepsis, obesity, and hypertension [29]. Uzawa also reported that the in vivo GCX thickness of 20-μm thick arteries was approximately 4 μm [4]. The GCXI in this study was 4–6 μm (Fig. 4A, B). Therefore, the GCXI of small arteries (40–60 μm thick) observed in this study is likely to represent the actual in vivo GCX thickness.

GCX has various functions, including the regulation of coagulation and vascular permeability, transduction of chemical signals, transportation of biochemical substances in and out of cells, and the maintenance of electrochemical barriers [31, 32]. Various factors, such as barrier size, barrier charge, and concentration gradient, are intricately related to vascular permeability, and it was reported as “revised Stirling’s law” because it considers the structure of GCX [33]. We focused on barrier size [34], including the “large pores and small pores” system. We used TMR-DEX40 (40 kDa), which is slightly larger than small pores but smaller in molecular weight than large pores, as a tracer of vascular permeability during endothelial dysfunction. Since HES130, which is gradually degraded by amylase [35], may be inappropriate as a tracer for vascular permeability, we prepared FITC-HES130 labeled with different fluorescent dyes from TMR and administered both simultaneously to investigate the behavior of HES130 during massive hemorrhage, and captured these changes over time.

The fluorescence intensity of TMR-DEX40 in the interstitial tissue among the NS-ALB and HES-NS groups was not significantly different after 60 and 90 min compared to that in group C (Fig. 7). The fluorescence intensities of FITC-HES130 in the NS-HES and HES-NS groups in the interstitial tissue were not significantly different at any time point when compared with that of group C (Fig. 8). HES130 administration had a strong effect on maintaining FITC-HES130 in the vessel lumen. Unlike DEX40, which is difficult to degrade in plasma, HES130 is gradually degraded. The HES-NS group had a lower interstitial fluorescence intensity than the two tracers, suggesting that the group HES130-NS may have maintained even smaller molecules, such as the degraded material of HES130, that pass through the small pore system in the vessel lumen.

In a report by Uzawa et al. [4], the interstitial distribution of FITC-HES70 was suppressed by HES130 administration, and the same tendency was observed in our study. Furthermore, this effect may have been enhanced by the prior administration of HES130. It was also reported that FITC-HES70 is localized on the inner wall of the vessel [4]. In this study, FITC-HES130 localization on the vessel inner wall was observed in intravital microscope images acquired 30 min after HES130 administration (Fig. 9, Online Resources 3–6). However, in the group with HES130, no such localization was observed, and it was uniformly present in the vessel lumen (Fig. 9). We speculated that this was probably due to HES130 already being attached to the vessel wall due to HES pre-administration and that FITC-HES130 was subsequently unable to attach to the vessel wall. In the immunoelectron microscopy images, there is a very intense positive reaction in the HES-NS group (Fig. 10F). Considering this electron microscopy result, the early binding of HES130 to the vascular endothelium of the impaired GCX is enhanced by prior administration of HES130.

We made the novel observation, in a murine model of acute hemorrhage, that HES130 has an affinity for the endothelial cell surface layer, also known as the GCX, and this affinity is enhanced by prior administration of HEZ130 by electron microscope. This animal study suggests that prior administration of HES130 during acute hemorrhage is beneficial. Localization of HES130 on the vessel wall may have a direct protective effect on the GCX or a sealing action on the vessel endothelial surface, which may also be considered as physical protection against an impaired GCX. This study may be insufficient to accurately clarify the effect of HES130 on the small pore system and its protective effect on GCX.

In terms of GCXI, the plasma concentration of syndecan-1, and the cumulative seven-day mortality rate, group NS-NS showed worse results than that of group C across all parameters. The syndecan-1 immunostaining images showed little staining of the inner vessel wall in the NS-NS group. Similar to previous reports [36], resuscitation with only saline during massive hemorrhage is likely to induce GCX damage and worsen the prognosis. However, the administration of colloidal solutions would be beneficial.

Numerous reports on the effects of HES130 in patients with massive hemorrhage [4, 37–39] have shown that its administration shortens the recovery time for shock [40]; improves peripheral circulation and vascular permeability; and reduces the concentration of GCX degradation products (syndecan-1, heparinase, and hyaluronic acid) [7]. In a clinical trial of HES130 administration in trauma patients, improvements in the base excess and lactate levels have also been reported [41]. Prior HES130 administration may help improve endothelial function by inhibiting vascular hyperpermeability, improving prognosis, and reducing the risk of circulatory failure as it localizes on the GCX.

Our study has three main limitations. First, we did not compare the efficacy of HES and albumin. The results of our study provide only one therapeutic strategy: prior administration of HES130 for initial fluid resuscitation in acute severe hemorrhage to prevent hyperpermeability due to endothelial dysfunction. Since albumin exists as a component of ESL [31], the fluorescent labeling of albumin is necessary to elucidate its mechanism of action and compare its relationship with HES130. Second, the side effects of HES, such as renal dysfunction, coagulation disorders, and allergies, were not investigated. We were unable to measure blood pressure; therefore, we do not know the actual duration or severity of shock in our study. Lastly, it is possible that the infusion volume in the NS-NS group was lower than that in the other groups regarding the volume expansion effect. However, considering the context-sensitive volume effect [42] and crystalloid fluid volume of large clinical studies [43, 44], which is approximately 1.2 times that of colloid fluid, the fluid volume in the NS-NS group was not extremely small in the very acute phase immediately after shock. In our research, we focused on the effect of rapid fluid resuscitation in the early stage of massive hemorrhage; experiments were conducted wherein approximately 60% of the whole blood volume was withdrawn. In this situation, irrespective of the type of infusion, most fluid solutions are expected to remain inside the vessels at an early stage of massive hemorrhage owing to a context-sensitive effect. Therefore, we decided to administer all resuscitation infusions in the same volume as the blood loss volume. However, because all mice were able to drink freely, their relative infusion volume may have been insufficient compared to that in the colloid infusion group during the early recovery period from shock in the NS-NS group. Therefore, the findings of this study may not be directly applicable to clinical practice.

## Conclusion

Saline administration as an initial fluid resuscitation method caused GCX impairment, increased vascular permeability, and worsened prognosis in a mouse model of acute massive hemorrhage. However, although colloid fluid therapies suppressed the leakage of syndecan-1, their prior administration preserved GCX thickness and improved prognosis. Especially, it was suggested that prior HES130 administration protects endothelial cell function by adhering to the vascular endothelial GCX.

## Supplementary Information

Below is the link to the electronic supplementary material.

Supplementary file1 (PDF 3814 KB)

Supplementary file2 (DOCX 31 KB)

Supplementary file3 (MPG 1196 KB)

Supplementary file4 (MPG 598 KB)

Supplementary file5 (MPG 1304 KB)

Supplementary file6 (MPG 984 KB)

Supplementary file7 (PDF 20 KB)

Supplementary file8 (DOCX 14 KB)

## Data Availability

All data generated or analyzed during this study are included in this published article and its supplementary information files.

## Abbreviations

NS: Normal saline
ALB: Albumin
HES: Hydroxyethyl starch
HES130: Hydroxyethyl starch 130/0.4/9
GCX: Glycocalyx
GCXI: Glycocalyx Index
FITC-HES130: Fluorescein isothiocyanate-labeled hydroxyethyl starch
TMR-DEX40: Tetramethylrhodamine-labeled dextran
FITC-WGA: Fluorescein isothiocyanate-labeled wheat germ agglutinin
FDA: Food and Drug Administration
EMA: European Medicines Agency
BGA: Blood gas analyses
SAS: Statistical Analysis Software
ANOVA: One-way analysis of variance
