# Supplementary figures and images for: The effect of tetrastarch on the endothelial glycocalyx layer in early hemorrhagic shock using fluorescence intravital microscopy: a mouse model
Source: J Anesth. 2022 Nov 24;37(1):104–18. doi: 10.1007/s00540-022-03138-4 (PMC9870981; doi:10.1007/s00540-022-03138-4)

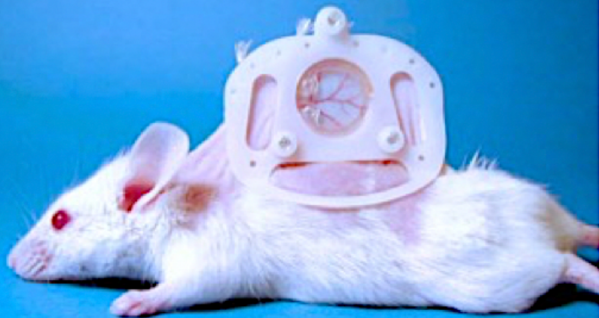

Supplement: Supplementary file 1 — Supplementary file1 (PDF 3814 KB) [file 540_2022_3138_MOESM1_ESM.pdf]

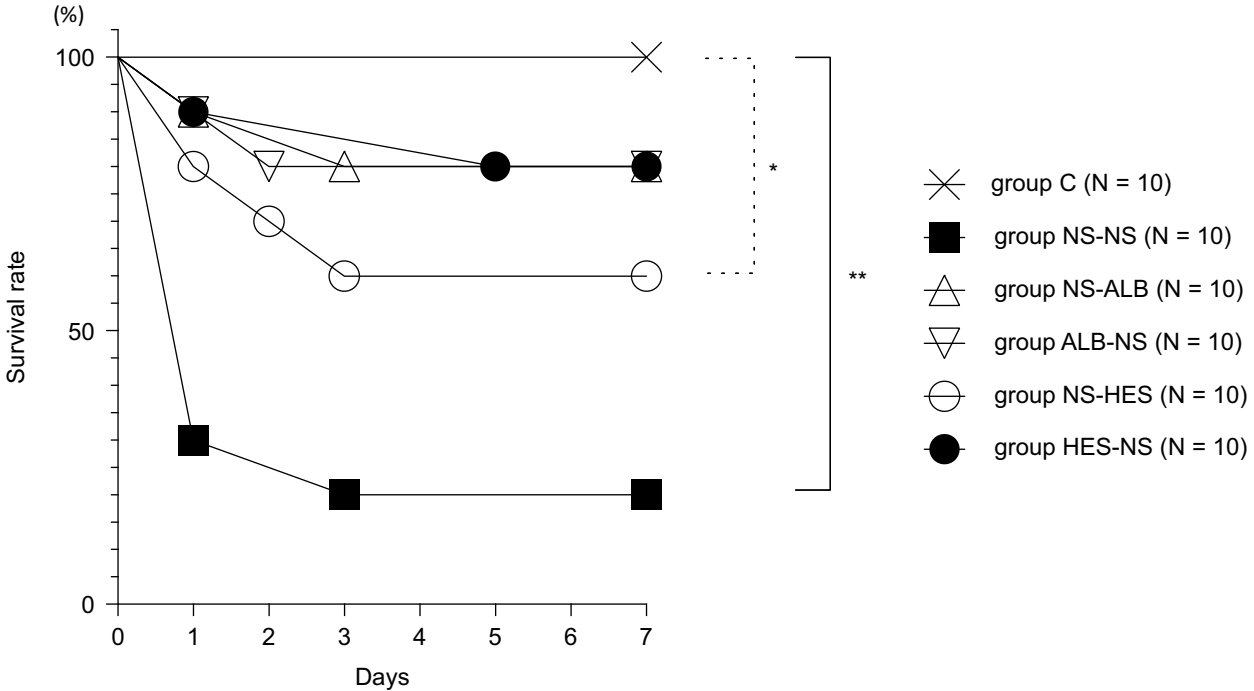

Supplement: Supplementary file 7 — Supplementary file7 (PDF 20 KB) [file 540_2022_3138_MOESM7_ESM.pdf]
