# Supplementary material for: The effect of tetrastarch on the endothelial glycocalyx layer in early hemorrhagic shock using fluorescence intravital microscopy: a mouse model
Source: J Anesth. 2022 Nov 24;37(1):104–18. doi: 10.1007/s00540-022-03138-4 (PMC9870981; doi:10.1007/s00540-022-03138-4)
Supplement: Supplementary file 2 — Supplementary file2 (DOCX 31 KB) [file 540_2022_3138_MOESM2_ESM.docx]

**The effect of tetrastarch on the endothelial glycocalyx layer in early hemorrhagic shock using fluorescence intravital microscopy: a mouse model**

**Journal**: *Journal of Anesthesia*

Tadao Ando^1^, Kohji Uzawa^1*^, Takahiro Yoshikawa^1^, Mitsuda Shingo^1^, Yoshihiro Akimoto^2^, Tomoko Yorozu^1^, Akira Ushiyama^3^

^1^Department of Anaesthesiology, Kyorin University School of Medicine

Mailing address: 6-20-2 Shinkawa, Mitaka-shi, Tokyo 181-8611, Japan

Tel: +81-43-261-5111/ Fax: +81-43-261-2305

^2^Department of Anatomy, Kyorin University School of Medicine, Tokyo, 181-8611 Japan

Tel: +81-43-261-5111/ Fax: +81-43-241-5452

^3^Living environment Research Department, National Public Health Institute

Mailing address: 2-3-6 Minami Wakou Saitama 351-0197, Japan

Tel: +81-48-458-6111/ Fax: +81-48-469-1573

*Corresponding Author: Kohji Uzawa

Department of Anaesthesiology, Kyorin University School of Medicine, 6-20-2 Sinkawa, Mitaka, Tokyo, 181-8611, Japan

Tel: +81-422-47-5511

Fax: +81-422-43-1504

E-mail: [kohji.fentanyl@gmail.com](mailto:kohji.fentanyl@gmail.com)

**Online Resource 2 Average weeks and body weight of the mice**

| Groups | N | Weeks | | Body weight (g) | |
| --- | --- | --- | --- | --- | --- |
|  |  | Mean±SD | Median (range) | Mean±SD | Median (range) |
| The Glycocalyx Index **(40 μm diameter)** | | | | | |
| Group C | 7 | 13.3±1.38 | 14 (11–15) | 26.6±0.65 | 26.8 (25.8–27.5) |
| Group NS-NS | 7 | 13.1±9.90 | 13 (12–15) | 26.0±1.55 | 26.6 (23.8–27.7) |
| Group NS-ALB | 8 | 12.9±1.46 | 13 (10–15) | 25.7±0.95 | 25.65 (24.5–27) |
| Group ALB-NS | 8 | 11.8±1.16^¶¶^ | 11.5 (10–13) | 24.9±1.42 | 24.4 (23.2–27) |
| Group NS-HES | 8 | 13.4±1.46 | 14 (11–14) | 26.1±1.70 | 25.7 (24.2–28.7) |
| Group HES-NS | 6 | 14.3±0.82^§§^ | 14.5 (13–15) | 26.1±1.07 | 25.95 (24.4–27.2) |
| **GCXI (60 μm diameter)** | | | | | |
| Group C | 10 | 12.1±1.52 | 11 (11–15) | 26.5±1.06 | 26.5 (25.2–28.6) |
| Group NS-NS | 9 | 12.9±0.93 | 13 (12–15) | 26.7±1.22 | 27 (24.5–28.3) |
| Group NS-ALB | 8 | 12.8±1.04 | 12.5 (12–15) | 25.8±0.92 | 26 (24.4–27) |
| Group ALB-NS | 7 | 11.4±1.13 | 11 (10–13) | 26.0±1.41 | 26.3 (24–27.9) |
| Group NS-HES | 10 | 12.9±1.29 | 13.5 (11–14) | 25.7±1.71 | 25.2 (23.7–28.7) |
| Group HES-NS | 8 | 12.9±1.64 | 13.5 (11–15) | 26.4±1.22 | 26.15 (24.4–27.9) |
| **Plasma syndecan-1** | | | | | |
| Group C | 9 | 12.6±1.42^§§, † ,‡^ | 13 (11–15) | 28.2±1.57 | 28 (25.4–30.7) |
| Group NS-NS | 7 | 17.3±3.68^‖‖, *^ | 17 (13–22) | 27.2±1.32 | 26.7 (26.2–30.1) |
| Group NS-ALB | 5 | 18.4±0.55^‖‖, *^ | 18 (18–19) | 27.3±1.31 | 26.05 (25.3–28.4) |
| Group ALB-NS | 6 | 22.0±3.95 ^**, ‖‖, ¶¶^ | 23.5  (14–24) | 28.7±1.73 | 28.85 (25.6–30.5) |
| Group NS-HES | 6 | 11.0±1.90 ^††, ‡‡, §§^ | 11 (8–13) | 27.5±1.57 | 26.05 (25.3–28.4) |
| Group HES-NS | 8 | 14.4±4.31^§§^ | 12 (10–21) | 26.6±1.46 | 27.6 (25.2–29.4) |
| **Fluorescence intensity (TMR-Dex40) in the interstitial space** | | | | | |
| Group C | 7 | 11.3±1.25^¶^ | 11 (10–13) | 26.8±1.68 | 26.3 (25–29.4) |
| Group NS-NS | 7 | 12.2±2.73 | 12 (9–16) | 26.5±1.37 | 26.7 (24.4–28.2) |
| Group NS-ALB | 7 | 11.6±1.27^¶^ | 11 (10–14) | 26.7±1.65 | 26.1 (24.2–28.8) |
| Group ALB-NS | 7 | 11.1±1.07^¶^ | 11 (10–13) | 25.7±0.96 | 25.3 (25–27.8) |
| Group NS-HES | 7 | 13.6±1.27 | 14 (12–15) | 26.2±0.87 | 26.5 (24.9–26.9) |
| Group HES-NS | 7 | 14.4±2.23 ^*, ‡, §^ | 14 (12–15) | 27.4±1.66 | 27.3 (25–29.9) |
| **Fluorescence intensity (FITC-HES130) in the interstitial space** | | | | | |
| Group C | 7 | 11.3±1.25^¶^ | 11 (10–13) | 26.8±1.68 | 26.3 (25–29.4) |
| Group NS-NS | 7 | 12.1±2.73 | 12 (9–16) | 26.5±1.37 | 26.7 (24.4–28.2) |
| Group NS-ALB | 7 | 11.6±1.27^¶^ | 14 (12–15) | 26.7±1.65 | 26.1 (24.2–28.8) |
| Group ALB-NS | 7 | 11.1±1.07^¶^ | 11 (10–13) | 25.7±0.96 | 25.3 (25–27.8) |
| Group NS-HES | 7 | 13.6±1.27 | 14 (12–15) | 26.2±0.87 | 26.5 (24.9–26.9) |
| Group HES-NS | 7 | 14.4±2.23^*, ‡, §^ | 13 (12–18) | 27.4±1.66 | 27.3 (25–29.9) |
| **Blood gas analysis** | | | | | |
| Group C | 10 | 13.3±3.37 | 12 (10–19) | 26.4±0.96 | 26.25 (25.3–27.9) |
| Group NS-NS | 8 | 15.1±3.36 | 16 (9–19) | 28.0±1.57 | 27.95 (25.5–30.3) |
| Group NS-ALB | 8 | 13.5±1.41 | 13.5 (12–15) | 27.4±1.47 | 27.7 (25.3–29.6) |
| Group ALB-NS | 7 | 17.7±4.79 | 16 (11–24) | 27.8±1.18 | 28.4 (25.6–28.8) |
| Group NS-HES | 8 | 14.0±2.00 | 14.5 (11–16) | 28.2±1.03 | 28.45 (26.7–29.5) |
| Group HES-NS | 8 | 15.3±3.99 | 14 (10–21) | 26.8±2.27 | 27.6 (22–29.1) |
| **Seven-day cumulative mortality rate** | | | | | |
| Group C | 10 | 13.6±1.51^¶¶, ‡^ | 13 (12–16) | 26.0±1.24 | 25.9 (24–27.7) |
| Group NS-NS | 10 | 12.9±2.51^‡‡, ¶¶^ | 12 (11–18) | 27.7±2.14 | 28.05 (23.2–31) |
| Group NS-ALB | 10 | 17.1±1.45^††, ‖‖, *^ | 17 (14–19) | 27.7±1.75 | 28.1 (24.8–31.2) |
| Group ALB-NS | 10 | 15.4±4.58^¶¶^ | 13 (13–24) | 26.6±0.76 | 26.58 (25.5–27.7) |
| Group NS-HES | 10 | 12.4±2.41^‡‡, ¶¶^ | 12 (11–19) | 25.9±1.62 | 25.3 (24.2–30) |
| Group HES-NS | 10 | 19.9±1.52 ^**, ††, §§, ‖‖^ | 19.5 (18–23) | 27.8±1.19 | 27.55 (26.2–29.7) |
| **Immunohistochemical staining of the endothelial surface layer** | | | | | |
| Group C | 1 | 16.0 |  | 28.5 |  |
| Group NS-NS | 1 | 15.0 |  | 29.8 |  |
| Group NS-ALB | 1 | 15.0 |  | 28.6 |  |
| Group ALB-NS | 1 | 15.0 |  | 28.2 |  |
| Group NS-HES | 1 | 15.0 |  | 27.8 |  |
| Group HES-NS | 1 | 15.0 |  | 28.7 |  |

Values are shown as the mean ±standard deviation and median (range). Groups NS-NS (normal saline [NS]→NS), NS-HES (NS→HES130), HES-NS (HES130→NS), NS-ALB (NS→albumin), and ALB-NS (albumin→NS), and group C (control, no withdrawal and no infusion). Data were obtained by one-way analysis of variance (ANOVA) followed by the Tukey-Kramer multiple comparison test. ** *P*≤0.01 and * *P*≤0.05. vs group C, ††*P*≤0.01 and † *P*≤0.05. vs group NS-NS, ‡‡ *P*≤0.01 and ‡ *P*≤0.05. vs group NS-ALB, §§ *P*≤0.01 and § *P*≤0.05. vs group ALB-NS, ‖‖ *P*≤0.01 and ‖ *P*≤0.05. vs group NS-HES, ¶¶ *P*≤0.01, ¶ *P*≤0.05. vs group HES-NS.
