# Supplementary material for: The effect of tetrastarch on the endothelial glycocalyx layer in early hemorrhagic shock using fluorescence intravital microscopy: a mouse model
Source: J Anesth. 2022 Nov 24;37(1):104–18. doi: 10.1007/s00540-022-03138-4 (PMC9870981; doi:10.1007/s00540-022-03138-4)
Supplement: Supplementary file 8 — Supplementary file8 (DOCX 14 KB) [file 540_2022_3138_MOESM8_ESM.docx]

**Online Resource 1. Dorsal skinfold chamber**

The DSC frame was surgically implanted between the two polyacetal resin frames. One side of the double layer of the dorsal skin was removed, and a cover glass was placed over the exposed side of the blood vessels. The cover glass was fixed using a ring. During the surgical implantation of the DSC chamber frames, mice were anaesthetised by intraperitoneal injection of ketamine (90 mg/kg body weight) and xylazine (10 mg/kg body weight). Experiments and observations were performed ≥1 week after the surgery to avoid inflammatory effects of the surgery and to stabilise the condition of the mice.

**Online Resource 3-6. Movie of Localization of FITC-HES130 and TMR-DEX40**

Representative movies at 30 min after blood removal, infusion treatment, and administration of fluorescent dyes (FITC-HES130 and TMR-DEX40) in the NS-NS and HES-NS groups. Online Resource movie 3 and 4 were acquired 30 min after the administration of FITC-HES130. FITC-HES130 was localized to the intravascular wall surface in the NS-NS group (Online Resource movie 3). It was uniformly distributed and not localized on the vascular endothelial surface in the HES-NS group (Online Resource movie 4). In contrast, Online Resource movie 5 and 6 were acquired 30 min after the administration of TMR-DEX40. Fluorescent TMR-DEX40 uniformly stained the intravascular space, including the endothelial surface, in both groups (Online Resource movie 5 and 6).

**Online Resource 7. Seven-day cumulative survival rate**

The cumulative survival rate over 7 days was measured and compared with that of group C (n=10 for each group). Data were analysed using the Kaplan-Meier method for 7-day survival and the log-rank test. ***P*≤0.01, **P*≤0.05.
